# Safety and efficacy of human amniotic membrane plug transplantation in cases of macular hole. A scoping review

**DOI:** 10.1186/s40942-024-00600-1

**Published:** 2024-10-25

**Authors:** Miguel A. Quiroz-Reyes, Erick A. Quiroz-Gonzalez, Miguel A. Quiroz-Gonzalez, Virgilio Lima-Gomez

**Affiliations:** 1 The Retina Department. Oftalmologia Integral ABC (Medical and Surgical Nonprofit Organization), National Autonomous University of Mexico, Lomas de Chapultepec, Av. Paseo de las Palmas 735 Suite 303, Mexico City, 11000 Mexico; 2 Juarez Hospital, Public Assistance Institution (Nonprofit Organization), Av. Politecnico Nacional 5160, Colonia Magdalena de las Salinas, Mexico City, 07760 Mexico

**Keywords:** Human amniotic membrane, Macular holes, Retinal detachment, Visual outcomes, hAM plug

## Abstract

**Background:**

Recently, there has been a surge of literature utilizing the human amniotic membrane (hAM) to manage cases of macular holes. In this scoping review, we aimed to systematically narrate the literature to identify cases of macular holes that are managed using hAM and explore the visual and anatomical outcomes to inform future research questions.

**Methods:**

This scoping review followed the Preferred Reporting Items for Systematic Reviews and Meta-Analyses guidelines. A detailed database search strategy (Scopus, Embase, Medline, and Cochrane Central) was developed to identify English-language published articles that reported using hAM to manage macular holes. All human clinical studies were included for a narrative data synthesis divided across study types.

**Results:**

The database search identified 82 articles, of which 34 were eligible for full-text review (0 randomized controlled trials (RCTs), 12 non-RCTs, 10 retrospective reviews, ten published case reports, and two clinical trial registries). The non-RCTs included patients with macular holes related to a wide range of retinal diseases, including retinal detachment, recurrent holes, and high myopia. Only two non-RCTs reported comparative data with a control group, but the study characteristics differed, and quantitative synthesis was impossible. Most retrospective interventional series and individual case reports reported a success rate of 93 -100% in hole closure and improvement in best-corrected visual acuity. None of the studies reported adverse effects after a hAM transplantation.

**Conclusion:**

The hAM effectively seals macular holes without any safety concerns, improving anatomical and visual outcomes in all macular holes.

**Supplementary Information:**

The online version contains supplementary material available at 10.1186/s40942-024-00600-1.

## Introduction

The human amniotic membrane (hAM) is the innermost layer of fetal membranes, with cells useful for regenerative medicine. It has been used in ophthalmology for several decades, with the earliest use reported in 1940, when it was used to repair conjunctival defects [1]. Because hAM has anti-inflammatory and pro-healing effects, its use has increased in the last few decades to treat corneal diseases [2] and macular holes [3].

Macular holes (MHs) cause central vision loss, particularly in older people. In Olmsted County, Minnesota, the reported incidence rates of idiopathic MHs are between 7.8 persons and 8.69 eyes per 100,000 people per year [4]. The disease, considered untreatable until the early nineties, has undergone different surgical interventions to improve visual outcomes [5]. Initially, vitrectomy with long-acting gas and a postoperative face-down position for at least one week was the only option. Over time, several variations and additions to the initial technique have been introduced, including tamponade, internal limiting membrane (ILM) peeling, combined lens surgery, and surgical adjuncts [6]. The success rates of different interventions for MHs depend on several prognostic predictors. Preoperative MH size is the most significant risk factor for surgical failure [7]. Surgeons use various tamponades to improve outcomes and face-down posturing. However, a Cochrane review concluded that for MHs ≤ 400 μm, face-down posturing had no significant effect on successful hole closure [8]. ILM peeling has increased anatomical and functional success rates in MH management. However, various consequences have been previously described, such as reduced retinal sensitivity and an increased incidence of perifoveal microscotomas [9]. Several advances in surgical techniques and equipment, including small-gauge vitrectomy surgery, limited vitrectomy, and posterior hyaloid face separation, have been made to improve visual function in MHs.

Recently, there has been a surge in the use of hAM to manage cases of MHs. In this context, we aimed to explore the literature to identify instances of MHs systematically managed via hAM and to explore visual and anatomical outcomes [6]. This is a descriptive analysis of the literature to determine the technique’s usefulness and identify the knowledge gap.

## Methods

This scoping review established eligibility criteria according to the PRISMA (Preferred Reporting Items for Systematic Reviews and Meta-Analyses) guidelines [10].

### Study eligibility criteria

Studies were selected for inclusion based on the prespecified population, intervention, comparison, and outcome (PICO) framework (Table 1). The aim was to include all clinical studies conducted with human participants, such as randomized controlled trials (RCTs) and nonrandomized, retrospective, prospective, or individual case reports. Animal studies, review articles, systematic reviews, and editorials were excluded.

**Table 1:** PICO criteria for the inclusion of studies

Parameter	Study selection criteria
Population	Human clinical studies (patients of all ages) that investigated the effectiveness of hAM in managing all types of MHs
Intervention	hAM grafting
Comparator	Traditional strategies used to manage MHs
Outcome measure (s)	Primary outcome(s): Postoperative BCVA (logMAR) at 6 weeks

BCVA, best-corrected visual acuity; hAM, human amniotic membrane; logMAR, logarithm of the minimum angle of resolution; MHs, macular holes

**Fig. 1:**
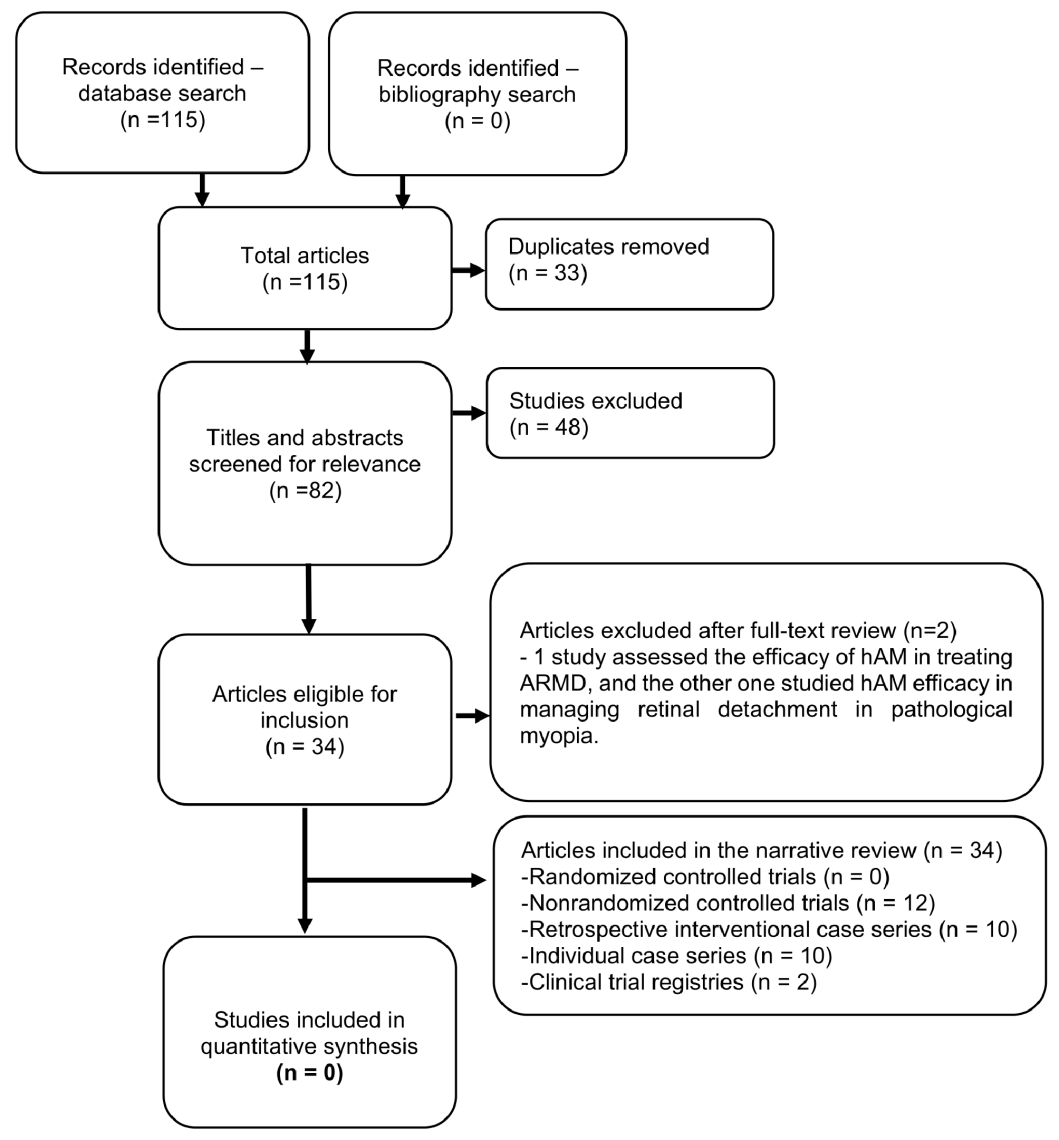
Study selection process

All clinical studies that reported the safety and efficacy of hAM in managing all types of MHs were eligible for inclusion. Study participants of any age group who underwent hAM graft transplantations were included. Studies were eligible if they used hAM as the primary or secondary intervention. Studies that combined hAM with other surgical procedures but did not report individual outcomes were excluded. Nevertheless, studies in which data related to hAM intervention could be recognized were included.

The comparison or control group in the study could receive usual care with traditional surgical or non-hAM interventions. Only studies reporting clinically relevant outcomes, such as visual acuity and anatomical outcomes, and only studies published in peer-reviewed English journals were considered eligible.

### Search strategy

The following electronic bibliographic databases were searched: Medline, PubMed, Embase, Scopus, and the Cochrane Central Register of Controlled Trials for studies published between 1980 and 07 June 2024. In all these databases, specific keywords were used to narrow the results to the desired literature. The bibliographies of the included articles were also searched to identify further relevant studies. The search strategy is described in the supplementary information file.

### Study selection process and data extraction

The bibliographic formats of the identified records in each database were first imported into Endnote Referencing software and then exported and uploaded to Covidence. This software automatically deduplicates records and facilitates double-anonymized title/abstract screening, full-text review, and data extraction. Two independent reviewers (MAQR and EAQG) screened the titles and abstracts, sought disagreements, and reached a consensus through collaborative discussions. The full texts of the records marked as potentially eligible were sought for retrieval. Two independent reviewers (MAQG and VLG) further screened the retrieved full-text articles for eligibility, and disagreements were resolved via consensus and collaborative discussions. Figure 1 summarizes the study selection process. The following data were extracted from the individual studies: authors, title, journal of publication/source, study design, country, sample size, participants’ inclusion and exclusion criteria, intervention characteristics, reported outcomes, and critical findings. One reviewer extracted the data, and a second reviewer confirmed the correctness of the data extracted by the first reviewer.

### Data narration and interpretation

Data from all studies were narrated by identifying similarities and differences between studies [11, 12]. This approach generated themes, combining closely related themes to form more abstract themes. The studies were then divided based on type (case reports, retrospective studies, prospective studies, and non-RCTs) and tabulated. The aim was to conduct a meta-analysis if more than two studies reported similar outcomes. However, the literature search did not retrieve any RCTs, and other studies varied in reporting outcome measures, as expected, and could not be meta-analyzed.

## Results

Through a database search for articles published between 1980 and 07 June 2024, 115 papers were identified (Scopus = 41, Embase and Medline = 70, and Cochrane Central = 4), and 33 duplicates were identified. The total number of eligible articles and abstracts was 82. After title and abstract review, 38 articles were eligible for full-text review. There were zero RCTs published on this topic. There were ten published case reports, ten retrospective reviews, 12 non-RCTs, and two clinical trial registries [13, 14]. Two studies were excluded after a full-text review, as the population of interest differed. One study assessed the efficacy of hAM in treating ARMD [15], and another studied hAM efficacy in managing retinal detachment in pathological myopia [16].

### Non-RCTs

Twelve non-RCTs were prospectively designed to evaluate the efficacy of the hAM graft in MH closure. They included participants with MHs related to retinal detachment [17, 18], recurrent MHs with previous interventions [19–25], and MHs due to high myopia [26, 27, 18]. Among all non-RCTs, two [25, 28] presented data as a comparative study. In one study, comparisons were made to assess the efficacy of PPV with the hAM plug against PPV with ILM flap insertion [28]. The authors suggested that the hAM plugging technique can achieve anatomical reduction and functional recovery of the retina in MHs. They reported that in the PPV with the hAM plug group, the holes were closed in five of seven eyes, and in the PPV with the ILM group, the MHs were closed in eight of nine eyes [28]. In another comparative non-RCT, the same group of researchers reported data comparing hAM plugs with 20% SF_6_ and air tamponade. The detailed clinical characteristics of the selected non-RCTs are shown in Table 2. The patients in both groups underwent similar surgical interventions, with the only difference being tamponade. They reported that the final BCVA recorded at 12 months was slightly better in the 20% SF_6_ tamponade group; however, the difference was not statistically significant [25]. Most non-RCTs reported visual acuity at six months, and most patients in these studies achieved complete MH closure after hAM insertion. All non-RCTs except two [28, 29] reported data as a single-arm interventional case series; no control group was included. Therefore, a quantitative analysis was not possible. The outcome measures differed in the two comparative studies, and a meta-analysis was impossible. Overall, the hAM plug obtained anatomical and visual success in over 90% of cases.

**Table 2:** Nonrandomized controlled trials

Study	Study design	Study details	Presenting BCVA	Procedure done	Final outcomes
Caporossi T et al.; 2022 [17]	Prospective, consecutive, nonrandomized interventional study.	19 eyes with MH retinal detachment who had undergone vitrectomy with ILM peeling, mean age was 63.8 ± 10.3 years,	The mean preoperative BCVA was 2 ± 1 logMAR (20/2000),	3-port 23-gauge PPV with hAM transplantation	12 months: mean BCVA: 1.1 ± 0.5 logMAR (Snellen equivalent of 20/250), MH closure was obtained in 94.7% (18 of 19 cases).
Caporossi T et al.; 2020 [18]	Prospective, consecutive, nonrandomized study	10 eyes with recurrent high myopic MHs associated with retinal detachment	Mean BCVA: 1.73 ± 0.4 logMAR	3-port 23-gauge PPV with hAM transplantation	6 months: 0.94 ± 0.23 logMAR, 100% MH closure
Qiao G et al.; 2022 [19]	Prospective nonrandomized case series	23 eyes with recurrent MHs who had undergone PPV with ILM peeling.	Mean BCVA: 1.73 ± 0.32 logMAR	25-gauge PPV, a hAM and C_3_F_8_ tamponade.	6 months: 1.12 ± 0.42 logMAR, MHs closed in 100%, no serious complications occurred.
C Wang et al.; 2024 [28]	Nonrandomized controlled clinical study, high myopia MH retinal detachment treated either with hAM plug or ILM flap insertion [full text in Chinese language]	PPV with hAM plug group: 7 eyes PPV with ILM flap insertion group: 9 eyes	N/A	PPV with hAM plug or PPV with ILM flap insertion	PPV with hAM plug group: MH closed in 5 of 7 eyes PPV with ILM insertion group: MH closed in 8 of 9 eyes The hAM plugging technique can achieve not only anatomical reduction but also functional recovery of the retina.
Caporossi T et al.; 2020 [27]	Prospective, consecutive, nonrandomized study	16 patients (mean age: 66.3 ± 8.4 years) with a recurrent high myopic MH that already underwent PPV with ILM peeling and endo tamponade.	Mean BCVA: 0.94 ± 0.24 logMAR	3-port 23-gauge PPV with hAM transplantation	Mean BCVA: 0.67 ± 0.26 logMAR, MH closed in 93.7% of eyes (15 of 16 eyes)
Rizzo S et al.; 2019 [20]	Prospective, interventional, consecutive case series	14 patients with recurrent MHs	Mean BCVA: 1.48 ± 0.49 logMAR	3-port 23-gauge PPV with hAM transplantation	Mean BCVA at 6 months: 0.71 ± 0.37 logMAR, no adverse events recorded.
Moharram HM et al.; 2020 [26]	Single arm prospective study	14 patients (average age: 58.7 years) with myopic MHs	Mean BCVA: 2.2 logMAR	PPV with ILM peeling and hAM graft	Mean BCVA at 6 months: 1.38 logMAR, no adverse events recorded.
Ahmad KH et al.; 2022 [21]	Prospective study	29 patients (mean age: 58 ± 6 years) with giant refractory MH	Mean BCVA: 1.54 ± 0.53	ILM peeling and hAM graft	Mean BCVA at 6 months: 0.84 ± 0.32, no adverse events recorded.
Hao Chen et al.; 2023 [22]	Prospective, interventional, and consecutive case series	12 patients (mean age: 63.3 ± 7.9 years) with unclosed MHs in previous surgeries.	Mean BCVA: 1.47 ± 0.58 logMAR	3-port 23-gauge PPV with hAM transplantation	Postoperative mean BCVA: 1.17 ± 0.60 logMAR, MHs remained closed.
Caporossi T et al.; 2021 [25]	Prospective interventional comparative study	hAM plug with 20% SF_6_ endotamponade: 10 eyes (mean age: 67 years) hAM plug with air as endotamponade: 10 eyes (mean age: 69 years)	hAM plug with 20% SF_6_ endotamponade: mean BCVA: 1.31 logMAR hAM plug with air as endotamponade: mean BCVA: 1.13 logMAR	3-port 23-gauge PPV with hAM transplantation	hAM plug with 20% SF_6_ endotamponade: final BCVA at 12-month: 0.53 logMAR hAM plug with air as endotamponade: final BCVA at 12-month: 0.55 logMAR
Garcin T et al.; 2022 [23]	Prospective interventional case series	10 patients (mean age of 62 ± 9 years) with at least one prior surgery involving ILM removal and intraocular tamponade.	Mean BCVA: 1.92 ± 0.58	3-port 23-gauge PPV with hAM transplantation	Mean BCVA at 12-month: 1.17 ± 0.57 logMAR, BCVA improved in 9 and worsened in 1 of 10 eyes, respectively.
Saad SM et al.; 2021 [24]	Quasi experimental	13 eyes with recurrent MH	Mean BCVA: 1.7 ± 0.33	hAM plug using pars plana approach	Mean BCVA: 0.9 ± 0.15, anatomic closure attained in 100% of cases

BCVA, best-corrected visual acuity; C_3_F_8_. octafluoropropane; hAM, human amniotic membrane; ILM, internal limiting membrane; MH, macular hole; N/A, not applicable; PPV, pars plana vitrectomy; SF_6_ sulfur hexafluoride

### Retrospective studies

Of the ten retrospective studies, two studies had two groups. One study [29], , compared PPV with autologous ILM transplantation and PPV with hAM plug transplantation in terms of BCVA at six months. There were three patients in each group. Another comparative study [30] was designed correctly, and the outcomes were appropriately reported. In a survey conducted by Yadav et al. [30], the safety and efficacy of the hAM plug (hAM group) were compared with those of inverted ILM peeling (control group) in MHs. They reported visual acuity data at two weeks, whereas most other studies reported BCVA data at six months. This retrospective interventional series [30] reported that 100% of patients in the hAM group and 80% in the control group achieved MH closure. Visual acuity improved by 0.1 logMAR in eight of the ten patients in both groups, and no complications were noted. The detailed clinical characteristics of the retrospective studies are shown in Table 3. No significant difference was found between the hAM plug and control groups regarding visual or anatomical responses. These studies suggest that hAM is an effective method for sealing MHs without any safety concerns.

**Table 3:** Retrospective case series

Study	Type	Purpose	Study details	Presenting BCVA	Procedure done	Final outcomes
Lee J et al.; 2023 [52]	Retrospective case report	To report the efficacy of hAM placement in cases of persistent MHs.	10 patients with full-thickness MHs	Mean BCVA was 1.6 logMAR (20/800)	Persistent full-thickness MHs treated with hAM.	**1 month** - Mean BCVA was 1.3 logMAR (20/400) **3 and 6 month** – mean BCVA was 1.1 logMAR (20/250) MHs remained closed in all cases until their last follow up at 6 months and no adverse effects reported.
Yadav NK et al.; 2020 [30]	Retrospective interventional case series	To compare the safety and efficacy of hAM plug in patients treated with hAM plug and those with inverted ILM peeling	**hAM group** 10 patients with hAM plugging for MHs, 7 idiopathic, 1 traumatic, and 1 patient each with MH induced retinal detachment and combined retinal detachment **Control group** 10 cases with similar configuration and duration of MHs treated with inverted peeling of the ILM	**hAM group**: Mean age: 62 ± 15.9 BCVA: 0.98 ± 0.3 logMAR **Control group**: Mean age: 67.6 ± 4.6 BCVA: 0.95 ± 0.22 logMAR	3-port, 25-gauge transconjunctival pars plana vitrectomy and hAM plug transplantation in the subretinal space under the MH	**hAM group**: 2-week BCVA: 0.81 ± 0.29 logMAR **Control group**: 2-week BCVA: 0.91 ± 0.31 **4 weeks**: 100% of cases in the hAM group achieved hole closure, and 80% of cases in the control group achieved hole closure. VA improved by 0.1 logMAR in 8 of 10 patients. No complications were noted. No significant difference was found between the hAM plug group and controls in visual and anatomical responses.
Abouhussein MA et al.; 2020 [54]	retrospective, interventional, consecutive case series	To evaluate the efficacy of hAM in promoting closure of MHs coexisting with rhegmatogenous retinal detachment.	14 eyes, mean age 63.58 ± 5.69 (52 to 71), MHs coexistent with peripheral retinal breaks,	1.87 ± 0.31 logMAR	The amniotic membrane patch was positioned in the MH under perfluorocarbon.	**6-month**: 0.67 ± 0.17 logMAR, all patients showed complete retinal reattachment with MH closure.
Bamberger MD et al.; 2022 [53]	Retrospective cohort study	To report on the use of hAM for MHs	22 patients with persistent or chronic MHs, median age of 61 years, MHs with a median size of 716 μm	Median BCVA 20/340	The hAM was introduced into the posterior segment using 23-gauge ILM forceps	Mean BCVA 20/370, the closure rate was 91% overall and ranged from 67–100% depending on the subtype of MHs.
Ferreira MA et al.; 2021 [55]	Retrospective chart review	To report the anatomical and functional results of off-label hAM graft as a primary intervention to repair large to giant MHs and in reoperations when wide ILM peeling was unsuccessful.	19 eyes, mean age = 66.2 ± 14.9 years,	Median BCVA 1.30 ± 0.44 logMAR (20/400)	The hAM was used to repair large to giant MHs.	Median BCVA 1.0 ± 0.72 logMAR, ~ approximately 20/200 with a median of three lines of VA, MHs resolved in 100% of patients at 9-month follow-up.
Lorenzi U et al.; 2022 [56]	Retrospective multicenter study	To evaluate the surgical management, outcomes and prognostic factors of full-thickness MHs without residual ILM using different surgeries, including hAM plug	58 eyes treated with hAM, mean age = 66 ± 12 years	Mean BCVA: 1.21 ± 0.45 logMAR	The hAM was positioned inside the hole, preferably with the basal membrane facing upward and the chorion oriented toward the RPE	0 Mean BCVA: 0.70 ± 0.34 logMAR, Full-thickness MHs closed in 93% of cases (53/58 cases)
Huang Yu H et al.; 2020 [57]	Retrospective interventional case series	To evaluate the surgical outcomes of cryopreserved and dehydrated hAM graft transplantation for MHs and MH retinal detachment	17 patients, mean age = 62.1 ± 10.0 years,	Mean BCVA: 1.38 ± 0.62 logMAR	23-gauge 3-port microincision vitrectomy and hAM graft transplantation	6 months: 76.5% (13 of 17) had sealed MHs, and the final BCVA among the improved cases was 1.12 ± 0.47 logMAR
Tsai DC et al.; 2020 [58]	Retrospective interventional case series	To report the surgical outcome and postoperative hypopigmented change around fovea among patients with high myopia who received hAM graft transplantation for MH.	10 eyes, mean age: 61.5 ± 8.4 years	Mean BCVA: 1.26 ± 0.48 logMAR	23-gauge PPV and hAM graft plug	Mean BCVA: 1.11 ± 0.44 logMAR, 70% had complete closure, and parafovea atrophy, a rare complication in the conventional MH surgery, was observed in 40% of eyes with highly myopic MHs after hAM graft transplantation.
Caporossi T et al.; 2020 [46]	Retrospective, consecutive, non-randomized interventional study	To report the anatomical and functional outcomes in a large series of patients affected by failed MHs and treated using a hAM plug.	36 patients (mean age: 66.3 ± 12.3 years) with failed MH	Mean BCVA: 1.15 ± 0.14 logMAR	23 or 25-gauge PPV and hAM graft plug	Mean BCVA at 6 months: 0.65 ± 0.26 logMAR; no adverse events were recorded.
Pacini B et al.; 2021 [20]	Retrospective study	To report the outcome of hAM transplant in failed MHs	PPV with autologous ILM transplant: 3 patients PPV with hAM plug transplant: 3 patients	PPV with autologous ILM transplant: Mean BCVA: 0.9 logMAR PPV with hAM plug transplant: Mean BCVA: 1.0 logMAR	PPV with autologous ILM transplant: 3 patients PPV with hAM plug transplant: 3 patients	PPV with autologous ILM transplant at 6 months: 0.7 logMAR PPV with hAM plug transplant at 6 months: 0.6 logMAR

BCVA, Best corrected visual acuity; hAM, human amniotic membrane; ILM, internal limiting membrane; logMAR, logarithm of the minimum angle of resolution; PPV, pars plana vitrectomy, RPE, retinal pigment epithelium; VA, visual acuity

### Case reports

There were ten case reports, which included patients of different age groups and sexes and a wide range of pathologies, including refractory MHs [31, 32], idiopathic MHs [33–35], giant MHs associated with Alport syndrome [32, 36], those associated with ARMD [37] and postraumatic [38], and pathological myopia [39]. These studies suggest that applying the hAM significantly improves the chances of MH closure. In addition, there was an initial improvement in visual acuity, but it tended to deteriorate over time, eventually stabilizing. The range of visual acuity achieved across these studies, depending on the initial visual acuity, was between 0.7 logMAR and 1.0 logMAR. These studies reported BCVA at one week, ten days, three weeks, four weeks [33], one month at [37], and six months at [34, 40]. These studies suggest that individual outcomes after hAM transplantation are highly successful. However, these data differ in terms of outcome reporting. The detailed clinical characteristics of the patients included in the case reports are shown in Table 4.

**Table 4:** Case reports

Study	Type	Purpose	Study details	Presenting BCVA	Procedure done	Final outcomes
Chalam KV et al.; 2024 [32]	Case report	To report the successful closure of a refractory giant MH (15 sq. mm) with hAM graft with an improvement in visual acuity	40/M, refractory MH, previous failed attempts of surgical repair	HM (right eye)	23-gauge PPV with hAM graft (4 mm x 4 mm)	BCVA: One month - CF at one foot One year – 20/200
Ventre L et al.; 2020 [35]	Case report	To investigate the outcome of hAM plug (diameter of 1.5 mm) in a MH (657 μm diameter)	70/F, idiopathic MH, treated with PPV and ILM peeling with gas tamponade, large MH	1.0 logMAR VA Pre-Op	23-gauge PPV with a hAM plug of 1.5 mm diameter	No change in VA in 1 week, 4 weeks, 0.9 logMAR at 6 weeks and 10 weeks.
Iannetta D et al.; 2024 [33]	Case report	To describe a new surgical technique involving the use of a hAM epiretinal patch to treat a primary MH retinal detachment in a highly myopic patient.	60/M, Primary MH treated with hAM patch	CF	PPV and ILM peeling and a patch of 1.5 mm diameter hAM over the macula	Four weeks after surgery, the macular hole closed, and the retina was reattached; 9-months of BCVA was 0.7 logMAR, and no postoperative adverse events were registered during the follow-up
Francois-Philippe R et al.; 2024 [36]	Case report	To describe the surgical management of bilateral giant full-thickness MH in a patient with Alport syndrome.	57/F, severe bilateral visual loss two months after cataract surgery, bilateral giant full-thickness MH.	20/160 in the right eye and 20/200 in the left eye.	25-gauge PPV with a hAM graft and gas tamponade	3 weeks: 20/50 in the right eye and 20/100 in the left eye, but vision deteriorated to 20/400 in both eyes two months after surgery. There was likelihood of the hAM graft contributing to preventing full-thickness MH progression by lowering the hole edges and filling the space between the two edges.
Siotto-Pintor E et al.; 2023 [37]	Case report	To report a case of a recurrent MH and atrophic ARMD treated hAM transplant.	72/M, recurrent MH and atrophic ARMD	20/400	25-gauge PPV with a hAM graft and gas tamponade	At 1-month, MH closed completely with BCVA improving to 20/320. However, after 1 year, the macular atrophic area increased, and the BCVA worsened to 20/400.
Lipkova B et al.; 2022 [31]	Case report	To evaluate the efficacy of vitrectomy, ILM peeling and a hAM insertion into MH.	Persistent MH in 3 patients, previous failed PPV surgeries	0.20, HM and 0.10	ILM peeling, hAM plug was inserted via MH subretinally	Two patients achieved MH closure; in the third patient, MH remained open, but the procedure reduced cystoid macular edema of the MH edges. There was an improvement in VA and a loss of disturbing visual phenomena.
Caporossi T et al.; 2019 [38]	Case report	To assess the efficacy of hAM to close a chronic postraumatic MH.	A 971 μm diameter MH	20/400 (1.3 logMAR)	PPV with hAM plug in the MH and SF_6_ as endo tamponade.	10 days: BCVA was 20/200 (1 logMAR), and the MH was closed. 3 months: BCVA improved to 20/100 (0.7 logMAR), and the MH remained closed. No adverse events were registered during the follow-up period.
Yin MY et al.; 2023 [39]	Case report	To treat pathological myopia and MH with hAM plug	60/F, with pathological myopia and MH retinal detachment, previously had vitrectomy surgery and ILM tamponade	HM	Treated with combined hAM tamponade silicone oil filling	VA improved to 0.05 from HM, the retina repositioned well, and MH closed, no severe complications were observed.
Proenca H et al.; 2020 [40]	Case report/Pictures and perspectives	To report the efficacy of hAM in long-standing refractory MH	48/F with long-standing refractory MH	20/400 (1.3 logMAR)	Treated with vitrectomy, subretinal cryopreserved hAM perfluoro propane endo tamponade and positioning	Six months: 20/200 (1.0 logMAR)
Baradad-Jurjo MC et al.; 2024 [34]	Case report	To describe a case of MH repaired using a subretinal hAM plug.	71/M, full-thickness MH in left eye	-	23-gauge PPV with ILM peeling and implantation of a subretinal hAM plug	Six months after the surgery, the hAM plug was completely integrated into the retina.

ARMD, age-related macular degeneration; BCVA, best-corrected visual acuity; CF, counting fingers; hAM, human amniotic membrane; HM, hand movements; ILM, internal limiting membrane; logMAR, logarithm of the minimum angle of resolution; MH, macular hole; PPV, pars plana vitrectomy, SF_6_, Sulphur hexafluoride; VA, visual acuity

## Discussion

This scoping review aimed to provide a descriptive overview of hAM transplantation’s usefulness in MH cases. It is a novel technique, and most scientific evidence comes from the last five years, from 2019, and only 115 papers mentioned this technique in managing MHs, as identified through our comprehensive literature search strategy. This review identifies the potential of hAM graft transplantation in MHs of varying origins for a successful hole closure. Interestingly, the hole closure rate reported across individual case studies, retrospective studies, and non-RCTs was more than 95%.

The treatment of recurrent MHs remains challenging despite the emergence of various techniques, such as the use of autologous lens capsules [41], free autologous ILM [42], and neurosensory retina autograft [43]. Despite developing multiple methods for managing MHs, a recurrent MH hole affects between 4.8 and 9.2% of individuals [26]. The current scoping review aimed to summarize and interpret the existing literature descriptively to formulate new research questions. We found that this technique has only been tried by a few institutions and is limited in geographical scope. Despite highly successful treatment outcomes, better-designed studies would be necessary to validate these findings further.

We must note that non-RCTs were found in our literature search. Therefore, the next logical step would be to design RCTs to examine the technique’s safety and efficacy. Based on the evidence presented in this review, ILM peeling could be a suitable point of comparison for hAM graft transplantation. It’s also clear that many conditions causing MHs were successfully treated. Therefore, after further investigation, hAM graft transplantation could be the graft of choice in cases of MHs.

Another observation noted in this review is the need for uniformity in reporting outcome measures. Most non-RCTs presented here were from the same research group and [20] used identical surgical methods, tools, and an outcome reporting format. To solidify the outcomes, the procedure must be tested by multiple researchers across the globe, which helps to stir scientific vigorousness [44]. For a meta-analysis in a systematic review, the outcome measures need to be reported in a similar format. From this review, the BCVA measured at six weeks and six months is the most conventional way of assessing visual outcomes [29, 45]. This is because the hAM will stabilize its location post-procedure, and the visual and anatomical outcomes will be comparable across studies.

The hAM graft has anti-inflammatory and antiangiogenic effects and secrets regenerative growth factors [46]. It acts as a scaffold for tissue reconstruction, supports surrounding cells, and limits their apoptosis. It inhibits the secretion of various inflammatory mediators such as interferon, interleukins, tissue necrosis, and platelet-derived growth factors [47, 48]. This is particularly important as recent evidence suggests that inflammation is the root cause of several retinal diseases [49], including MHs [50], and hAM transplantation may help to dampen inflammation. Using hAM transplantation, reconstruction of limbal stem cell deficient corneal surface has been tried before [2]. Experimental and clinical studies have previously shown that transplantation of corneal stem cells cultured on an amniotic membrane for corneal burns shows excellent results in terms of the reduction of stromal opacity and ocular inflammation [51]. Translating this evidence from the anterior segment of the eye to the posterior segment for treating recalcitrant MHs [52, 53], MHs that occur with RRD [54], large to giant MHs [55], refractory MHs [56], approaches using cryopreserved or dehydrated hAM graft types [57], and understanding potential consequences [58] is logical and needs further investigation.

One systematic review has recently published a single-arm meta-analysis evaluating the safety and efficacy of hAM in refractory MHs [3]. The review reported the findings of 8 studies on 103 eyes that had undergone failed vitrectomy and ILM peeling. Their analysis suggested a 66% chance of visual acuity improvement, a 94% chance of hole closure, and a 6% chance of graft dislocation. They also indicated that cryopreserved hAM grafts might have better outcomes than dehydrated grafts. However, the limitation of this systematic review is the need for a control group, as discussed earlier. It warrants properly designed RCTs to evaluate the absolute safety and efficacy of hAM graft transplantation compared to established surgical procedures in cases of MH management.

## Conclusions

The current research on using the hAM graft technique for treating MHs is limited due to inconsistencies in the populations tested, variations in reported visual acuity measurements, time frames, and a mix of study populations with different diseases. The review included various types of published articles to provide comprehensive evidence. Non-RCTs and case series showed significant differences and could not be meta-analyzed, and no RCTs were available for meta-analysis. However, case studies and retrospective case series indicated a promising trend in using hAM plugs for treating MHs and demonstrated positive outcomes. Notwithstanding the mentioned limitations, this review identified the potential for the hAM graft technique as either an adjunctive therapy or an effective treatment for a wide range of MH cases.

## Electronic supplementary material

Below is the link to the electronic supplementary material.

Supplementary Material 1

## Data Availability

No datasets were generated or analysed during the current study.

## Abbreviations

ARMD: Age-related macular degeneration
BCVA: Best-corrected visual acuity
C_3_F_8_: Octafluoropropane
hAM: Human amniotic membrane
ILM: Internal limiting membrane
logMAR: The logarithm of the minimum angle of resolution
MHs: Macular holes
PICO: Population, intervention, comparison, outcomes
PRISMA: Preferred reporting items for systematic reviews and meta-analyses
PPV: Pars plana vitrectomy
RCTs: Randomized clinical trials
SF_6_: Sulfur hexafluoride
VA: Visual acuity
