# Supplementary material for: Safety and efficacy of human amniotic membrane plug transplantation in cases of macular hole. A scoping review
Source: Int J Retina Vitreous. 2024 Oct 25;10:82. doi: 10.1186/s40942-024-00600-1 (PMC11515266; doi:10.1186/s40942-024-00600-1)
Supplement: Supplementary file 1 — Supplementary Material 1 [file 40942_2024_600_MOESM1_ESM.docx]

**Supplementary information file**

Database search strategy

( TITLE-ABS-KEY ( macular AND holes ) OR TITLE-ABS-KEY ( retinal AND holes ) AND TITLE-ABS-KEY ( amniotic AND membrane ) OR TITLE-ABS-KEY ( amnion ) OR TITLE-ABS-KEY ( amnian AND plug ) OR TITLE-ABS-KEY ( amnion AND membrane ) AND TITLE-ABS-KEY ( amnion AND graft ) OR TITLE-ABS-KEY ( human AND amnion AND membrane OR ham ) ) AND ( LIMIT-TO ( DOCTYPE , "ar" ) ) AND ( LIMIT-TO ( EXACTKEYWORD , "Human" ) OR LIMIT-TO ( EXACTKEYWORD , "Retina Macula Hole" ) OR LIMIT-TO ( EXACTKEYWORD , "Amnion" ) OR LIMIT-TO ( EXACTKEYWORD , "Retina Tear" ) OR LIMIT-TO ( EXACTKEYWORD , "Retinal Perforations" ) OR LIMIT-TO ( EXACTKEYWORD , "Human Amniotic Membrane" ) OR LIMIT-TO ( EXACTKEYWORD , "Controlled Study" ) OR LIMIT-TO ( EXACTKEYWORD , "Vitreoretinal Surgery" ) OR LIMIT-TO ( EXACTKEYWORD , "Amniotic Membrane" ) OR LIMIT-TO ( EXACTKEYWORD , "Intervention Study" ) OR LIMIT-TO ( EXACTKEYWORD , "Tissue Graft" ) OR LIMIT-TO ( EXACTKEYWORD , "Lyophilized Human Amniotic Membrane" ) OR LIMIT-TO ( EXACTKEYWORD , "Amniotic Membrane Transplantation" ) OR LIMIT-TO ( EXACTKEYWORD , "Recurrent Macular Hole" ) OR LIMIT-TO ( EXACTKEYWORD , "Human Amniotic Membrane Graft" ) OR LIMIT-TO ( EXACTKEYWORD , "Amniotic Membrane Implant" ) )
